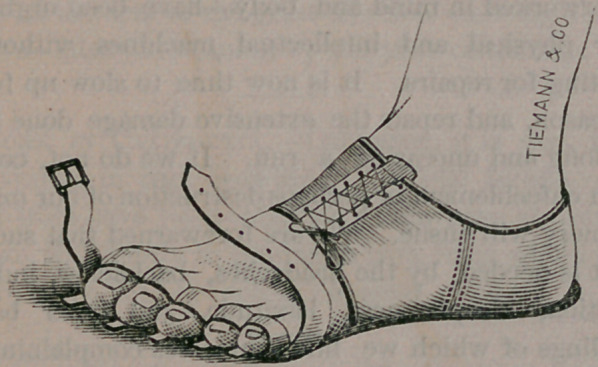# Deformities of the Toes

**Published:** 1875-04

**Authors:** 


### DEFORMITIES OF TOES.

One of the most painful affections of the feet is that produced by a large corn or hardened scale upon the second or prominent joint of the great toe. It is ordinarily designated as a *bunion*, and is produced by wearing a short, tight, and high-heeled boot, causing the entire weight of the body, in the act of walking, to be thrown upon the toes. The integument over the joint becomes hard and calloused, producing inflammation in the bone, which often results in a *bursa* or abscess, causing the toe to be turned inward, and thus producing a large and prominent protuberence at the joint. By reason of the tenderness of the joint, great pain is produced by the pressure of the boot or shoe, and the resulting deformity is often considerable.

To allay the inflammation, leeching, and local applications of acetate of lead, water, and opium should be applied.*

If an abscess is present, it should be freely opened and the matter set free. After the inflammation has subsided, the following apparatus can be applied, with a view to removing the deformity:

It consists of a light, well-tempered, lever of steel, with an oval ring in the centre and provided with hinges, to allow of free motion in the joint. The oval ring fits evenly and smoothly around the bunion, permitting no pressure upon its surface, and is attached to the instep by the steel spring and a piece of webbing, and to the great toe in a similar manner, as is fully shown in the engraving. The enlarged joint should be painted with tincture of iodine, once or twice daily. The apparatus can be worn under a loosely fitting boot or shoe, and should be continued until the toe has regained its

*R. Water, oz. 8; acetate of lead, dr. 1; laudanum, oz. 3. Mix.

normal position. Of course, it is understood that the tension of the spring is regulated by the lacings at the toe. The tighter these are drawn—without producing absolute pain—the more rapid will be the cure.

The same cause which gives rise to bunions, inflamed and enlarged joints, viz. tight and badly fitting boots and shoes, also produces over-riding toes. We have seen cases of this character where the toe formed such an object of prominence upon its fellow, as to entirely debar its owner from wearing anything but a large, baggy, and hideous looking boot. A sort of retribution, we imagine, for crowding said toes within the narrow limits of an Exquisite's tight, patent-leather boots.

This deformity of the toes becomes very painful and very much "in the way," so that we have been frequently consulted with a view to amputation of the offending member. A much better plan, and one that almost invariably results in returning the over-riding toe to the ranks with its fellows, is to apply an apparatus, here illustrated; and we give these very fine cuts because they convey a much better idea of what is intended, in a much smaller space, than pages of letter-press could accomplish.

This apparatus consists simply of a steel sole, arranged with spaces to correspond to the number of toes. A piece of soft webbing is then passed through these slits and over each and every toe, something after the plan of weaving, until every member is brought down upon a level with its fellow. The strap is then suitably fastened, and the appliance kept in position by a silk or cotton covering, cut to fit, and laced over the instep, as shown in the cut.

A shoe can be worn over the apparatus and, in a few weeks, or months, depending upon the severity of the case, the cure will be effected.

These appliances can be made sufficiently well by any one having mechanical ingenuity, but will give better satisfaction perhaps, to have them made by the regular instrument maker, the cost being trifling. By placing your bare foot upon a sheet of paper, and marking around it with a pencil, you can have an exact outline of the foot. Then give the ankle and instep measurement, as for a pair of shoes, and send them, with diagram, to Geo. Tiemann & Co., New York, or if you prefer, to the editor of this journal, and the articles can be ordered for you.

## Figures and Tables

**Figure f1:**
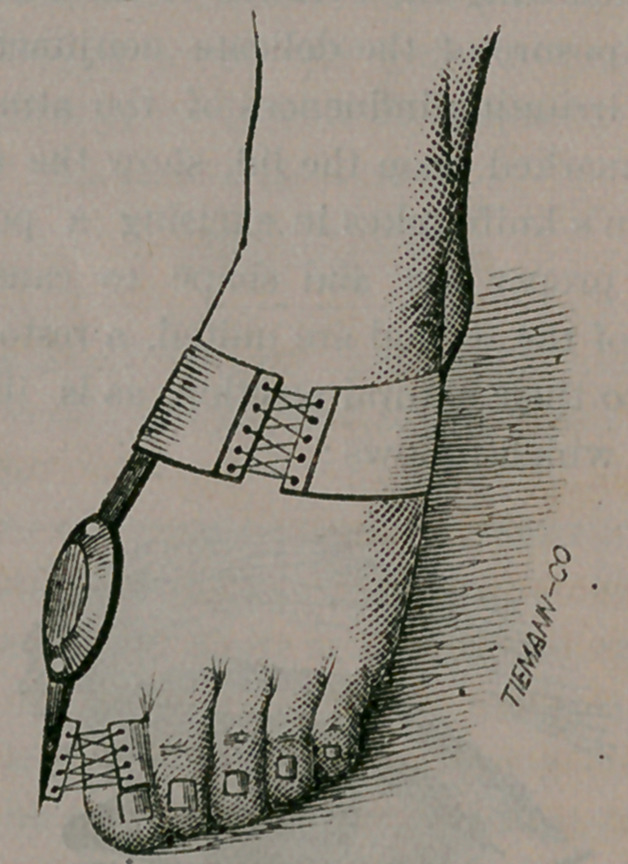


**Figure f2:**